# Interference-Robust Transmission in Wireless Sensor Networks

**DOI:** 10.3390/s16111910

**Published:** 2016-11-14

**Authors:** Jin-Seok Han, Yong-Hwan Lee

**Affiliations:** Department of Electrical and Computer Engineering and INMC, Seoul National University, Seoul 151-742, Korea; jshan1201@ttl.snu.ac.kr

**Keywords:** co-channel interference, low-power wireless sensor network, transmission scheme

## Abstract

Low-power wireless sensor networks (WSNs) operating in unlicensed spectrum bands may seriously suffer from interference from other coexisting radio systems, such as IEEE 802.11 wireless local area networks. In this paper, we consider the improvement of the transmission performance of low-power WSNs by adjusting the transmission rate and the payload size in response to the change of co-channel interference. We estimate the probability of transmission failure and the data throughput and then determine the payload size to maximize the throughput performance. We investigate that the transmission time maximizing the normalized throughput is not much affected by the transmission rate, but rather by the interference condition. We adjust the transmission rate and the transmission time in response to the change of the channel and interference condition, respectively. Finally, we verify the performance of the proposed scheme by computer simulation. The simulation results show that the proposed scheme significantly improves data throughput compared with conventional schemes while preserving energy efficiency even in the presence of interference.

## 1. Introduction

Recent advances in wireless communications and electronic device technology make it possible to realize low-power and low-cost wireless sensor networks (WSNs). The low cost of deployment of low-power WSNs can make various IoT services quite feasible [1]. However, low-power WSNs operating in unlicensed spectrum bands may seriously suffer from unavoidable co-channel interference generated by other radio systems (e.g., IEEE 802.11b/g/n wireless local area networks (WLANs)) [2,3,4,5]. It is of great concern to deploy low-power WSNs that can provide stable transmission performance in the presence of co-channel interference.

A number of techniques has been proposed for the coexistence of various radio systems operating in unlicensed spectrum bands. It is well recognized that WLAN is one of major interference sources that seriously hamper the operation of low-power WSNs [6,7]. A simple technique is to allocate WSN and WLAN devices spectrum resource orthogonal to each other or to exploit the utilization of white space with channel switching [8,9,10,11,12]. Recently, channel hopping mechanisms have been applied to low-power WSNs to mitigate the interference. For example, IEEE 802.15.4e deterministic synchronous multi-channel extension (DSME) employs a channel hopping and channel adaptation scheme for channel diversity [13]. Bluetooth low energy (BLE) employs an adaptive frequency hopping (AFH) mechanism [14]. However, these approaches may not be effective as the available spectrum is getting sparse due to the increase of active WLAN devices [15,16]. The performance of the AFH mechanism may considerably be degraded in the presence of multiple WLAN interference mainly due to its slow adaptation process [17]. Although WLAN devices employ a carrier sense multiple access with collision avoidance (CSMA/CA) scheme, they may not detect the presence of WSN signals whose transmission power is much lower than theirs [18]. As a consequence, they may continue the signal transmission even in the presence of low-power WSN signals, significantly deteriorating the operation of low-power WSNs. Some works have considered placing a special device to help low-power WSNs share a channel with WLANs. A special device, referred to as a signaler, may help other ZigBee devices to access the channel by sending a busy tone, forcing WLAN devices to defer their transmissions [18]. However, the coordination between the signaler and other ZigBee devices may be too complex, making it impractical in WLAN operation environments. Another special device, referred to as the arbitrator, can schedule the activity of ZigBee and WLAN devices through communication among them [19]. However, the scheduling may not be easy in dynamic interference environments, since it may require the arbitrator to re-initiate a spectrum scanning and re-allocate the parameters.

**Figure 1:**
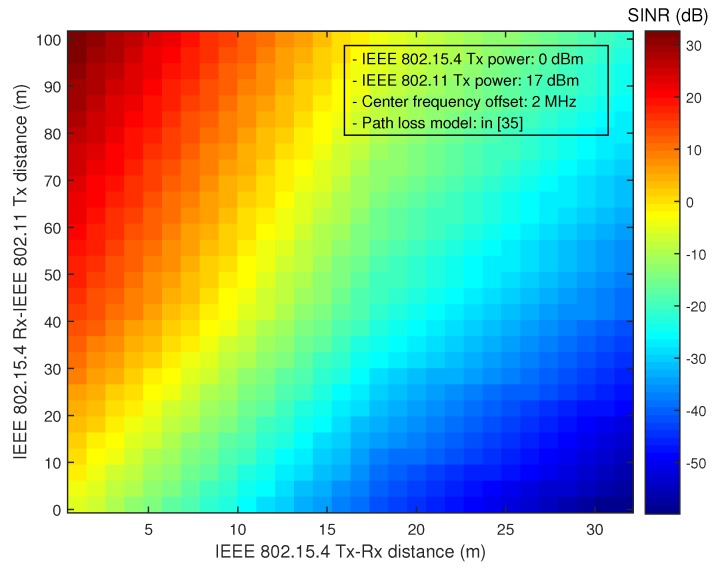
signal-to-interference-plus-noise ratio (SINR) of IEEE 802.15.4 in the presence of co-channel interference.

Some previous works have also considered performance improvement of low-power WSNs in the presence of co-channel interference. Two approaches, the collision-recovery and collision-avoidance approach, have mainly been considered. The collision-recovery approach mainly considers the use of forward error correction (FEC) techniques to mitigate the co-channel interference. For example, BuzzBuzz employs a Hamming(12,8) code-based FECscheme for ZigBee devices [20]. However, it may not be effective in dynamic interference environments. A real-time adaptive transmission (RAT) scheme makes WSN devices choose an appropriate FEC coding scheme to maximize the throughput [21]. However, these collision recovery schemes may not be effective unless the signal-to-interference-plus-noise ratio (SINR) is sufficiently high. Figure 1 illustrates the SINR of IEEE 802.15.4 in the presence of co-channel interference. Note that IEEE 802.15.4 requires a minimum SINR of 0.4 dB for the transmission of a 20-byte packet (refer to Appendix A). However, the SINR may not be high in practical operating environments, making the collision-recovery approach ineffective for reliable signal transmission.

The collision-avoidance approach mainly exploits white space to avoid co-channel interference. A dynamic rate adaptation and control for energy reduction (DRACER) scheme adjusts the transmission rate in response to the change of operating condition [22]. DRACER may reduce the probability of collision with the interference signal by transmitting packets at the highest rate. However, it does not consider the effect of the packet size in the presence of interference, yielding inefficient use of white space. A white space-aware frame adaptation (WISE) scheme considers the packet size control [15]. Modeling the idle period of interference as a Pareto-distributed random variable, WISE determines the packet size to maximize the throughput efficiency, while providing desired packet collision probability. However, it may need to periodically adjust the Pareto model through channel sensing, which may be a considerable overhead to low-power WSN devices. Another packet size control scheme, referred to as dynamic packet length control (DPLC), simply adjusts the packet size based on a transmission efficiency metric [23]. DPLC empirically determines the packet size after a number of packet transmissions. However, it may not properly work in the presence of time-varying interference. Moreover, these collision-avoidance schemes only consider the signal transmission at a fixed rate, which may not be efficient when the signal-to-noise ratio (SNR) is higher than that required for the rate. A payload size and transmission rate adjustment scheme was proposed for WLANs in slow fading channel environments [24]. However, it does not consider the presence of co-channel interference, making it impractical for application to low-power WSNs.

As a collision-avoidance approach, partial packet recovery (PPR) techniques were proposed to reduce the transmission of acknowledgment (ACK) packets. They partition a data packet into a number of smaller blocks and acknowledge the transmission of multiple data packets by a single recovery frame including a block map, which describes the status of all blocks [25,26,27,28]. The transmitter should retransmit all of the packets when the recovery frame is not received [25] or wait until it receives the recovery frame, while the receiver retransmits the recovery frame until it receives data packets [26,27,28]. The PPR techniques may severely suffer from frequent loss of recovery frames in practical operation environments. Moreover, they do not consider the waiting time when they evaluate the throughput and the energy consumption. The power consumption during the idle listening of WLAN and ZigBee devices is comparable to the power consumption during signal reception [29]. Moreover, these schemes only consider the transmission at a fixed rate.

In this paper, we consider the adjustment of the transmission rate and payload size to improve the transmission performance in the presence of co-channel interference. We consider the operation of low-power WSNs that can support the bulk transfer of large data (e.g., e-price tags [30], surveillance applications involving imaging/acoustics [31,32] and structural health monitoring [33,34]). Based on the probability of transmission failure and the data throughput, we determine the payload size to maximize the data throughput in the presence of interference. It is shown that the transmission time maximizing the normalized throughput is little affected by the transmission rate, rather mostly by the interference condition. We independently adjust the transmission time and the transmission rate to maximize the data throughput in response to the change of operation environments. Finally, we evaluate the performance of the proposed scheme by computer simulation.

The rest of this paper is organized as follows. Section 2 describes the WSN model in consideration. Section 3 analyzes the performance of WSN in the presence of co-channel interference. Section 4 describes the proposed scheme to improve the throughput performance in the presence of co-channel interference. Section 5 evaluates the performance of the proposed scheme by computer simulation. Finally, Section 6 concludes this paper.

## 2. System Model

**Figure 2:**
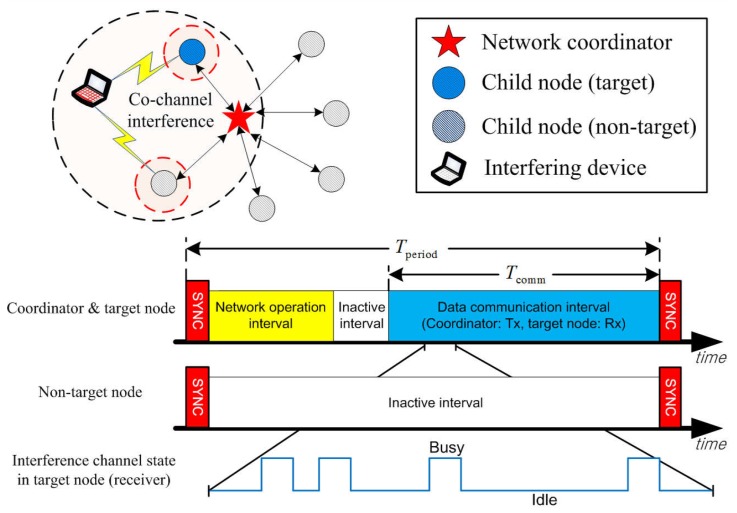
A star-topology WSN in a synchronized operation mode.

As illustrated in Figure 2, we consider a star-topology WSN comprising a network coordinator and its child nodes located in an operation range of WLANs. The WSN employs a periodic frame structure for synchronous network operation and data communications, where the period of the frame and the length of the data communication interval are $T_{\mathrm{period}}$ and $T_{\mathrm{comm}}$, respectively. The network coordinator transmits a beacon at the beginning of each frame for synchronized network operation. It allocates communication resources to a target node using a handshaking protocol during the network operation interval [35]. Non-target nodes may stay in sleep mode to minimize power consumption.

We assume that a transmitter node generates $L_{\mathrm{bulk}}$-bit data at each transaction. The $L_{\mathrm{bulk}}$-bit data is fragmented by a number of data packets each of which comprises a *L*-bit data payload ($L_{\min}\leq L\leq L_{\max}$) and signaling bits (e.g., packet header). The receiver confirms the packet reception by sending an ACK packet. The transmitter retransmits the data packet if it does not receive an ACK packet. We also assume that the transmission rate is adjustable according to the channel condition. Then, the packet transmission time with transmission mode *m* can be represented as:

(1) $$T_{\mathrm{pkt},m}\left(L\right)=\frac{L_{\mathrm{shr}}+L_{\mathrm{phr}}}{R_{\mathrm{base}}}+\frac{L_{\mathrm{mhr}}+L}{R_{m}}$$

where $L_{\mathrm{shr}}$, $L_{\mathrm{phr}}$ and $L_{\mathrm{mhr}}$ are respectively the bit size of the synchronization header (SHR), the physical layer packet header (PHR) and the medium access control (MAC) layer packet header (MHR), $R_{m}$ ($\in \left\{R_{1},R_{2},...,R_{M}\right\}\equiv \Pi$) denotes the transmission rate of MHR and data payload of transmission mode *m* and $R_{\mathrm{base}}$ is the transmission rate of SHR and PHR ($R_{\mathrm{base}}\in \Pi$). We assume that $R_{1}<R_{2}<\cdots <R_{M}$, and the ACK packet has no payload (i.e., L=0).

A node with transmission mode *m* may experience packet loss when the received SNR, denoted by γ, is lower than a threshold ${\hat{\gamma }}_{m}$ and/or when the packet has collision with the interference signal. We assume that the SNR is unchanged during each packet transmission and randomly varies between the packet transmissions [36]. Then, the probability of transmission failure can be represented as:

(2) $${\tilde{p}}_{m}\left(L,\gamma \right)=1-\left(1-p_{m,c}\left(L\right)\right)\left(1-p_{m,s}\left(L,\gamma \right)\right)$$

where $p_{m,c}$ and $p_{m,s}$ denote the probability of transmission failure due to the packet collision and low SNR, respectively. The probability of transmission failure due to SNR can be represented as:

(3) $$p_{m,s}^{}=1-\left(1-p_{\mathrm{fail}}^{\mathrm{sync}}\left(\gamma ,L_{\mathrm{shr}}\right)\right){\left(1-b_{m,s}^{}\left(\gamma \right)\right)}^{L_{\mathrm{phr}}+L_{\mathrm{mhr}}+L}$$

where $p_{\mathrm{fail}}^{\mathrm{sync}}\left(\gamma ,L_{\mathrm{shr}}\right)$ denotes the probability of synchronization failure at an SNR of *γ* when the SHR length is $L_{\mathrm{shr}}$ (e.g., $p_{\mathrm{fail}}^{\mathrm{sync}}\left(\gamma ,L_{\mathrm{shr}}\right)$ for IEEE 802.15.4 communications is referred to in [37]) and $b_{m,s}\left(\gamma \right)$ denotes the bit error rate (BER) of transmission mode *m* at an SNR of γ. Note that ${\tilde{p}}_{m}$ is an upper bound of the packet error rate (refer to Appendix B).

The probability of transmission failure due to the packet collision can be calculated in terms of the channel occupancy of the interference signal. The channel occupancy of the WLAN signal can be characterized using a semi-Markov model [38,39]. The channel has two simple operation states, the busy and idle state, whose duration is described by the probability density function (PDF) $f_{T_{\mathrm{busy}}}\left(t\right)$ and $f_{T_{\mathrm{idle}}}\left(t\right)$, respectively. The channel occupancy of the interference signal can be defined by:

(4) $$\rho =\frac{\tau_{\mathrm{busy}}}{\tau_{\mathrm{busy}}+\tau_{\mathrm{idle}}^{}}$$

where $\tau_{\mathrm{busy}}$ and $\tau_{\mathrm{idle}}$ denote the mean duration of the busy and the idle state, respectively. It can be shown that $p_{m,c}$ can be represented as:

(5) $$p_{m,c}=\rho p_{m,c|\mathrm{busy}}+\left(1-\rho \right)p_{m,c|\mathrm{idle}}$$

where $p_{m,c|\mathrm{busy}}$ and $p_{m,c|\mathrm{idle}}$ denote the probability of transmission failure due to the packet collision when the packet transmission is initiated in the presence and the absence of the interference signal, respectively. For a given probability of transmission failure, denoted by ${\tilde{p}}_{m,c}\left(L,\gamma \right)$, the data throughput can be represented as:

(6) $$S_{m}\left(L,\gamma \right)=\frac{L\left(1-{\tilde{p}}_{m,c}\left(L,\gamma \right)\right)}{{\tilde{T}}_{m}\left(L\right)}\;[\mathrm{bit}/s]$$

where ${\tilde{T}}_{m}$ denotes the data transaction time (i.e., the round trip time).

Define the bulk transfer delay $D_{\mathrm{bulk}}$ by the sum of the access delay $D_{\mathrm{acc}}$ and the transmission delay $D_{\mathrm{tx}}$. The access delay is the time difference between the start of an access attempt and the successful access. Then, it can be shown that:

(7) $$\begin{array}{ccc} D_{\mathrm{acc}} & = & \frac{1}{2}T_{\mathrm{period}}+\left(1-p_{\mathrm{sync}}\right)\left(1-p_{\mathrm{sch}}\right)T_{\mathrm{period}}\\ & & +\left\{p_{\mathrm{sync}}+\left(1-p_{\mathrm{sync}}\right)p_{\mathrm{sch}}\right\}\left(1-p_{\mathrm{sync}}\right)\left(1-p_{\mathrm{sch}}\right)2T_{\mathrm{period}}+\cdots \\ & = & \frac{1}{2}T_{\mathrm{period}}+\frac{1}{\left(1-p_{\mathrm{sync}}\right)\left(1-p_{\mathrm{sch}}\right)}T_{\mathrm{period}} \end{array}$$

where $p_{\mathrm{sync}}$ and $p_{\mathrm{sch}}$ denote the failure probability of the frame synchronization and the scheduling, respectively. Note that a low-power WSN can maintain the frame synchronization robustly in the presence of co-channel interference [40]. The transmission delay is the time difference between the beginning of data transmissions and the end of all $L_{\mathrm{bulk}}$-bit data transmissions. Then, it can be shown that:

(8) $$\begin{array}{c} D_{\mathrm{tx}}=\frac{1}{\left(1-p_{\mathrm{sync}}\right)}\left\lceil \frac{L_{\mathrm{bulk}}}{T_{\mathrm{comm}}\bar{S}}\right\rceil T_{\mathrm{period}} \end{array}$$

where $\bar{S}$ denotes the average data throughput. We maximize the average data throughput of low-power WSNs in the presence of co-channel interference by adjusting the transmission rate and the payload size, minimizing the transmission delay.

## 3. Transmission in the Presence of Interference

We estimate the probability of transmission failure and data throughput in the presence of interference and then determine the payload size maximizing the data throughput. We assume that a node transmits data packets without consideration of the channel condition and confirm the transmission by receiving an ACK packet. The data transaction time can be represented as:

(9) $$\begin{array}{c} {\tilde{T}}_{m}=T_{\mathrm{pkt},m}^{data}+T_{\mathrm{pkt},m}^{ack}+2\delta \end{array}$$

where $T_{\mathrm{pkt},m}^{data}=T_{\mathrm{pkt},m}\left(L\right)$, $T_{\mathrm{pkt},m}^{ack}=T_{\mathrm{pkt},m}\left(0\right)$ and *δ* denotes the time from the transmission to the reception state and vice versa. The probability of transmission failure due to the packet collision can be represented as:

(10) $$p_{m,c}=1-\left(1-p_{m,c}^{data}\right)\left(1-p_{m,c}^{ack}\right)$$

where $p_{m,c}^{data}$ and $p_{m,c}^{ack}$ denote the collision probability of data and ACK packets, respectively.

When a data packet is transmitted in the presence of interference, it may experience the collision with a probability of one (i.e., $p_{m,c|\mathrm{busy}}=1$). Even when it is transmitted in the absence of interference, it may probabilistically experience the collision. Although a WLAN device transmits the signal after carrier sensing-based clear channel assessment (CCA) or energy-based CCA, it may not detect the presence of the signal transmitted by low-power WSN devices. The probability of packet collision can be represented as:

(11) $$\begin{array}{c} p_{m,c}^{data}=\left\{\begin{array}{c} \rho ;\;\mathrm{if}\;\mathrm{int}.\;\mathrm{source}\;\mathrm{can}\;\mathrm{detect}\;\mathrm{data}\;\mathrm{packets}\\ \rho +\left(1-\rho \right)\;p_{m,c|idle}^{data};\;\mathrm{otherwise} \end{array}\right. \end{array}$$

where $p_{m,c|idle}^{data}$ can be derived in what follows.

For given idle state length $T_{\mathrm{idle}}$ and data packet transmission time $T_{\mathrm{pkt},m}^{data}$, a packet collision may occur if a data packet is transmitted ($T_{\mathrm{idle}}-T_{\mathrm{pkt},m}^{data}$) seconds after the beginning of the idle state. Let $t_{d}$ be the time difference between the beginning of the idle state and the presence of a new data packet (i.e., $0\leq t_{d}\leq T_{\mathrm{idle}}$). Assuming that the duration of the idle state is Pareto or exponentially distributed, it can be shown that:

(12) $$\begin{array}{ccc} p_{m,c|idle}^{data}\left(t_{d}\right) & = & \mathrm{Pr}\left[T_{\mathrm{idle}}<t_{d}+T_{\mathrm{pkt},m}^{data}|t_{d}\right]\\ & = & \left\{\begin{array}{c} 1-{\left(\frac{t_{d}}{t_{d}+T_{\mathrm{pkt},m}^{data}}\right)}^{\sigma };\;\mathrm{Pareto}\;\mathrm{dist}.\\ 1-\exp\left(-\tau_{\mathrm{idle}}^{-1}T_{\mathrm{pkt},m}^{data}\right);\;\exp.\;\mathrm{dist}. \end{array}\right. \end{array}$$

where *σ* denotes the shape parameter of Pareto distribution. It can be seen that the probability depends on $t_{d}$ when the idle state is Pareto-distributed. When the idle state is exponentially distributed, Equation (11) can be rewritten as:

(13) $$\begin{array}{c} p_{m,c}^{data}=\left\{\begin{array}{c} \rho ;\;\mathrm{if}\;\mathrm{int}.\;\mathrm{source}\;\mathrm{can}\;\mathrm{detect}\;\mathrm{data}\;\mathrm{packets}\\ 1-\left(1-\rho \right)\exp\left(-\tau_{\mathrm{idle}}^{-1}T_{\mathrm{pkt},m}^{data}\right);\;\mathrm{otherwise}. \end{array}\right. \end{array}$$

Similarly, it can be shown that the collision probability of an ACK packet after successful data packet transmission can be represented as:

(14) $$p_{m,c}^{ack}=1-\exp\left\{-\tau_{\mathrm{idle}}^{-1}\left(\delta +T_{\mathrm{pkt},m}^{ack}\right)\right\}.$$

The probability of transmission failure due to the packet collision can be represented as:

(15) $$p_{m,c}\left(L\right)=1-\left(1-\rho \right)\exp\left(-\frac{L+\alpha_{m}}{R_{m}\tau_{\mathrm{idle}}}\right)$$

where $\alpha_{m}$ is a constant indifferent from the payload size *L* and can be determined as:

(16) $$\begin{array}{ccc} \alpha_{m} & = & {\alpha^{\prime }}_{m}R_{m}\\ & = & \left(2T_{\mathrm{base}}+\frac{L_{\mathrm{mhr}}^{data}+L_{\mathrm{mhr}}^{ack}}{R_{m}}+\delta \right)R_{m}. \end{array}$$

Here, Tbase=Lshr+LphrLshr+LphrRbaseRbase, $L_{\mathrm{mhr}}^{data}$ and $L_{\mathrm{mhr}}^{ack}$ respectively denote the MHR bit size of the data and the ACK packet.

It can be shown that the data throughput can be represented as:

(17) $$S_{m}\left(L,\gamma \right)=R_{m}\frac{L}{L+\beta_{m}}\left(1-\rho \right)\exp\left(-\frac{L+\alpha_{m}}{R_{m}\tau_{\mathrm{idle}}}\right){\left(1-b_{\mathrm{base},s}\left(\gamma \right)\right)}^{L_{\mathrm{phr}}^{data}+L_{\mathrm{phr}}^{ack}}{\left(1-b_{m,s}\left(\gamma \right)\right)}^{L+L_{\mathrm{mhr}}^{data}+L_{\mathrm{mhr}}^{ack}}$$

where $\beta_{m}=\beta_{m}^{\prime }R_{m}=\left({\alpha^{\prime }}_{m}+\delta \right)R_{m}$ and $b_{\mathrm{base},s}$ denotes the BER of PHR transmission. Assuming that $b_{\mathrm{base},s}$ and $b_{m,s}$ are very low with the use of an appropriate transmission rate in the absence of interference, Equation (17) can be approximated as:

(18) $$S_{m}\left(L,\gamma \right)\approx R_{m}\frac{L}{L+\beta_{m}}\left(1-\rho \right)\exp\left(-\frac{L+\alpha_{m}}{R_{m}\tau_{\mathrm{idle}}}\right).$$

Taking the derivative of Equation (18) with respect to *L*, i.e.,

(19) $$\frac{\partial S_{m}}{\partial L}=-\frac{\tau_{\mathrm{idle}}^{-1}\left(1-\rho \right)}{{\left(L+\beta_{m}\right)}^{2}}\exp\left(-\frac{L+\alpha_{m}}{R_{m}\tau_{\mathrm{idle}}}\right)\left(L^{2}+\beta_{m}L-\beta_{m}R_{m}\tau_{\mathrm{idle}}\right)$$

we can see that there exists a payload size that maximizes the data throughput. This implies a tradeoff between the packet transmission efficiency and the probability of transmission failure. The use of a smaller payload size may improve the robustness to interference, but it may also increase the signaling overhead, deteriorating the overall transmission efficiency.

The payload size maximizing the data throughput can be determined as:

(20) $$\begin{array}{c} L_{m}^{*}≅-\frac{\beta_{m}}{2}+\sqrt{{\left(\frac{\beta_{m}}{2}\right)}^{2}+\beta_{m}R_{m}\tau_{\mathrm{idle}}}. \end{array}$$

It can be seen that the payload size depends on the average idle period of interference, $\tau_{\mathrm{idle}}$, and the transmission rate $R_{m}$, as well. With $\beta_{m}=\beta_{m}^{\prime }R_{m}$, Equation (20) can be rewritten as:

(21) $$\begin{array}{ccc} L_{m}^{*} & = & \left[-\frac{{\beta^{\prime }}_{m}}{2}+\sqrt{{\left(\frac{{\beta^{\prime }}_{m}}{2}\right)}^{2}+{\beta^{\prime }}_{m}\tau_{\mathrm{idle}}}\right]R_{m}\\ & = & T_{m}^{*}R_{m} \end{array}$$

where $T_{m}^{*}$ denotes the payload transmission time that maximizes the data throughput. It can be shown that:

(22) $$\begin{array}{c} \frac{\partial L_{m}^{*}}{\partial R_{m}}=T_{m}^{*}+R_{m}\frac{\partial T_{m}^{*}}{\partial R_{m}}>>\frac{\partial T_{m}^{*}}{\partial R_{m}}. \end{array}$$

Since $R_{m}$ is typically large, Equation (22) implies that the sensitivity of the payload transmission time with respect to the transmission rate is much smaller than that of the payload size. Moreover, $T_{m}^{*}$ depends on $\beta_{m}^{\prime }$. The sensitivity of $\beta_{m}^{\prime }$ with respect to the transmission rate is much smaller than that of $\beta_{m}$. The transmission time can approximately be represented as:

(23) $$\begin{array}{ccc} T_{m}^{v} & ≅ & -\frac{\beta_{}}{2}+\sqrt{{\left(\frac{\beta_{}}{2}\right)}^{2}+\beta \tau_{\mathrm{idle}}}\\ & = & T_{}^{*}. \end{array}$$

It can be seen that $T^{*}$ depends on $\tau_{\mathrm{idle}}$ and not on the transmission rate. Note that the payload size $L_{m}^{*}$ depends on the transmission rate.

## 4. Proposed Transmission Scheme

Exploiting the above investigation, we consider the performance improvement of low-power WSNs in the presence of interference. We determine the initial payload size based on the interference characteristics estimated by Equation (20). Exploiting Equation (23), we adjust the transmission rate *R* and the transmission time *T* in response to the change of channel and interference condition, respectively. Algorithm 1 summarizes the proposed scheme. 

**Algorithm 1:** Overall process of the proposed scheme 1:Initialize $R\leftarrow R_{\mathrm{init}}$ using $\tilde{\gamma }$ 2:Initialize $L\leftarrow L_{\mathrm{init}}$ using ${\tilde{\tau }}_{\mathrm{idle}}$ and *R* 3:$L\leftarrow \mathrm{median}\left(L_{\min},L,L_{\max}\right)$ 4:T←LLRR
 5:Initialize $I_{T}\leftarrow 1$ and ${\tilde{S}}_{\mathrm{new}},{\tilde{S}}_{\mathrm{old}}\leftarrow 0$ 6:**while** $L_{\mathrm{bulk}}$-bit data are not delivered **do** 7: **for** *i* = 1:*W* **do** 8:  Transmit a data packet with *R* and *L* 9:  **if** an ACK packet is received **then**10:   $n_{\mathrm{fail}}\leftarrow 0$
11:   S˜new←S˜new+TTT+β′T+β′
   Update *R* using $\tilde{\gamma }$13:   
$L\leftarrow \mathrm{median}\left(L_{\min},RT,L_{\max}\right)$
14:   
T←LLRR
15:   **else**16:   $n_{\mathrm{fail}}++$
17:   **if** $n_{\mathrm{fail}}>N_{\mathrm{fail}}$ **then**
18:    $R\leftarrow R_{1}$
19:    $L\leftarrow \mathrm{median}\left(L_{\min},RT,L_{\max}\right)$
20:    T←LLRR
21:   **end if**
22:  **end if**
23: **end for**24: Update *T* by **Algorithm 2**25: $L\leftarrow \mathrm{median}\left(L_{\min},RT,L_{\max}\right)$
26: T←LLRR
27:**end while**


We initially determine the payload size by estimating the average idle period of interference. We define the interference estimation interval by the dedicated interval within the first data communication interval of a pair of scheduled nodes. With the use of an energy-type detector for the channel sensing, the transmitter node can estimate the channel occupancy of interference signal as:

(24) $$\begin{array}{c} \tilde{\rho }=\frac{1}{N_{s}}\sum_{j=1}^{N_{s}}I\left\{y_{j}>\lambda \right\} \end{array}$$

where $I\left\{\cdot \right\}$ is an indicator function, $y_{j}$ is the energy of the *j*-th received sample, *λ* is a threshold level and $N_{s}$ is the total number of samples for the measurement. It can be shown that the average busy period of interference can be estimated as:

(25) $${\tilde{\tau }}_{\mathrm{busy}}=\frac{1}{N_{\mathrm{busy}}}\sum_{j=1}^{N_{\mathrm{busy}}}n_{\mathrm{busy},j}T_{s}$$

where $N_{\mathrm{busy}}$ is the number of busy periods, $n_{\mathrm{busy},j}$ is the length of the *j*-th busy period and $T_{s}$ is the channel sensing interval. Finally, the average idle period of interference can be estimated as:

(26) $${\tilde{\tau }}_{\mathrm{idle}}={\tilde{\tau }}_{\mathrm{busy}}\left(\frac{1}{\tilde{\rho }}-1\right).$$

As an example, when $N_{s}=10$ and $T_{s}=320$ us, assume that the result of channel sensing is {O,O,X,X,X,O,X,X,X,X}, where “O” and “X” denote the presence and the absence of interference, respectively. Then, it is estimated that the channel occupancy of interference signal is 0.3; the average busy period of interference is 480 usand the average idle period of interference is 1.12 ms, since $N_{\mathrm{busy}}=2$, $n_{\mathrm{busy},1}=2$ and $n_{\mathrm{busy},2}=1$. The initial payload size can be determined as:

(27) $$L_{\mathrm{init}}=-\frac{\beta_{m}}{2}+\sqrt{{\left(\frac{\beta_{m}}{2}\right)}^{2}+\beta_{m}R_{m}{\tilde{\tau }}_{\mathrm{idle}}}$$

where the initial transmission rate can independently be determined in what follows.

The transmitter node determines the transmission rate based on the estimated SNR. It determines the transmission rate of the next data packet based on the received signal strength (RSS) of the ACK packet received most recently. It can determine the initial transmission rate from the RSS of received packets. For an estimated SNR $\tilde{\gamma }$, it determines the transmission rate by the highest transmission rate $R_{m}$ that satisfies $\tilde{\gamma }\geq {\hat{\gamma }}_{m}$, where ${\hat{\gamma }}_{m}$ is the minimum SNR for transmission mode *m*, which can provide the desired packet error rate of $p_{s}$ with the use of maximum payload size in the absence of interference and can be represented by:

(28) γ^m=bm,s-11-ps11LmaxLmax.

Here, $b_{m,s}^{-1}$ denotes the inverse function of $b_{m,s}$. As described in Section 2, the packet loss can occur due to low SNR and/or packet collision. If the packet loss is mainly due to the low SNR, it may be desirable to decrease the transmission rate *R*. If it is mainly due to the packet collision, it may be desirable to decrease the transmission time *T* to reduce the collision probability.

Consider the case that the transmission failure consecutively occurs due to the packet collision, and the transmitter node decreases the transmission rate. Then, the transmission time will be increased, and thus, the packet collision problem may rather be exacerbated. This problem can be alleviated by adjusting the transmission rate and the transmission time together. If the number of consecutive transmission failures reaches a threshold $N_{\mathrm{fail}}$, the transmitter node reduces the transmission rate, while keeping the transmission time *T* unchanged. If the transmission rate is adjusted from $R_{a}$ to $R_{b}$, it may be desirable to adjust the payload size from $L_{a}$ to $L_{b}$ as:

(29) $$L_{b}^{}=R_{b}T_{}^{}=R_{b}\left(\frac{L_{a}^{}}{R_{a}}\right).$$

It may be feasible for the transmitter node to adjust the transmission time *T* according to the interference condition, while adjusting the transmission rate according to the channel condition. The normalized throughput, defined by S˜=SmSmRmRm, can be estimated by:

(30) $$\tilde{S}=\frac{T}{T+{\beta^{\prime }}_{}}\sum_{i=1}^{W}I\left\{\mathrm{ACK}\;\mathrm{packet}\;\mathrm{is}\;\mathrm{received}\;\mathrm{for}\;\mathrm{the}\;i-\mathrm{th}\;\mathrm{data}\;\mathrm{packet}\right\}$$

after performance measurement of *W*-packet transmission. Note that this metric is not affected by the adjustment of the transmission rate since it is normalized with respect to the transmission rate. After each *W*-packet transmission, the transmitter node updates the normalized throughput, say ${\tilde{S}}_{\mathrm{new}}$. Comparing ${\tilde{S}}_{\mathrm{new}}$ with a previous one, say ${\tilde{S}}_{\mathrm{old}}$, it can adjust the transmission time *T* to increase the normalized throughput. Let Δ be the step size for the adjustment of transmission time and $I_{T}\left(=\pm 1\right)$ be a parameter indicating whether the transmission time was increased or decreased previously. If ${\tilde{S}}_{\mathrm{new}}>\eta {\tilde{S}}_{\mathrm{old}}$, where η≥1, it implies that the transmission time was effectively adjusted. In this case, it may be desirable to keep the adjustment. The transmitter node increases or decreases the transmission time by Δ according to $I_{T}$. If ${\tilde{S}}_{\mathrm{old}}>\eta {\tilde{S}}_{\mathrm{new}}$, it implies that the previous adjustment was not effective, requiring the change of the sign of $I_{T}$ (i.e., $I_{T}\leftarrow -I_{T}$). Then, the transmitter node adjusts the transmission time by $I_{T}\Delta$. Otherwise, the transmitter node does not adjust the transmission time. It may also be desirable to change the step size in consideration of the difference between ${\tilde{S}}_{\mathrm{new}}$ and ${\tilde{S}}_{\mathrm{old}}$. If the difference is large, it may be desirable to use a larger step size to quickly adjust the transmission time. Algorithm 2 summarizes the adjustment of the transmission time.
**Algorithm 2:** Adjustment of transmission time 1:**if** ${\tilde{S}}_{\mathrm{new}}>{\tilde{S}}_{\mathrm{old}}>0$ **then**
 2: **if** ${\tilde{S}}_{\mathrm{new}}>\eta_{1}{\tilde{S}}_{\mathrm{old}}$ **then**
 3:  $T\leftarrow \Delta_{1}^{I_{T}}T$
 4: **else if** ${\tilde{S}}_{\mathrm{new}}>\eta_{2}{\tilde{S}}_{\mathrm{old}}$ **then** 5:  $T\leftarrow T+I_{T}\Delta_{2}$
 6: **end if**
 7:**else** 8: **if** ${\tilde{S}}_{\mathrm{old}}>\eta_{1}{\tilde{S}}_{\mathrm{new}}$ **then** 9:  $T\leftarrow \Delta_{1}^{-I_{T}}T$
10: **else if** ${\tilde{S}}_{\mathrm{old}}>\eta_{2}{\tilde{S}}_{\mathrm{new}}$ **then**
11:  $T\leftarrow T-I_{T}\Delta_{2}$
12: **end if**
13: $I_{T}\leftarrow -I_{T}$
14:**end if**
15:${\tilde{S}}_{\mathrm{old}}\leftarrow {\tilde{S}}_{\mathrm{new}}$ and ${\tilde{S}}_{\mathrm{new}}\leftarrow 0$

## 5. Performance Evaluation

**Figure 3:**
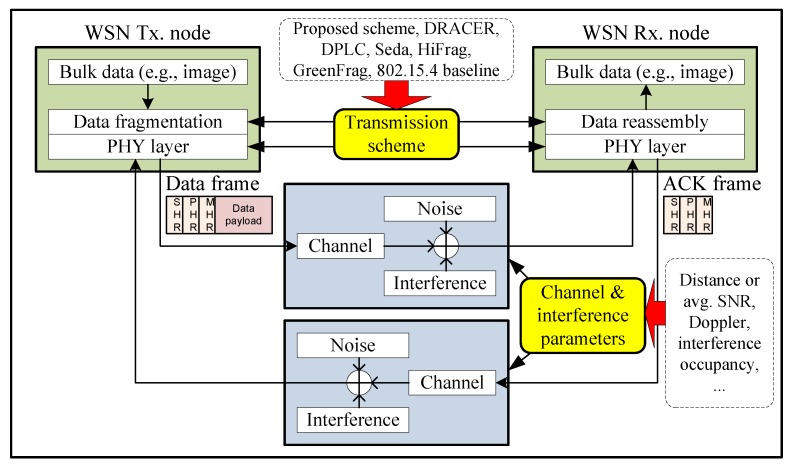
WSN simulator structure.

We evaluate the performance of the proposed scheme by computer simulation using a lab-developed WSN simulator written in C++. Figure 3 depicts the simulator structure, which considers data transmission from the network coordinator to its child nodes in the presence of IEEE 802.11g WLAN interference signals [38,39] in a Ricean fading channel with a maximum Doppler frequency of $f_{D}$ [36]. For the performance evaluation, we use Monte Carlo simulation of 300 iterations, each of which runs $1.5\times 10^{5}$ simulation time slots.

The simulation environment is summarized in Table 1, which is mainly based on the specification of the IEEE 802.15.4 PHY layer. For comparison, we also consider the performance of seven schemes; an IEEE 802.15.4 baseline scheme at a transmission rate of 250 Kbps with a fixed payload size, DRACER, which adjusts a transmission rate with a fixed payload size [22], DPLC, which adjusts a payload size at a fixed transmission rate of 250 Kbps [23], DRACER with DPLC that adjusts the transmission rate and the payload size by using DRACER and DPLC, respectively, a streaming data link layer scheme, which is a static PPR scheme referred to as Seda [25], a hybrid frame fragmentation scheme, which is a dynamic PPR scheme referred to as HiFrag [27], and a green frame fragmentation scheme, which is a combination of HiFrag and transmit power adaptation, referred to as GreenFrag [28]. The proposed scheme and DRACER use one of four transmission rates, 250, 500, 1000 and 2000 Kbps, by adjusting the spreading factor with an appropriate coding set, while using the same spectrum bandwidth as conventional IEEE 802.15.4 [22,41,42]. The transmission rate can be informed to the receiver using a start frame delimiter (SFD) without additional signaling overhead [22]. Considering application areas of WSNs, we assume that the maximum payload size is 1024 bytes, which is larger than that of the conventional IEEE 802.15.4 PHY layer (i.e., 127 bytes). Note that IEEE 802.15.4g, a recent amendment of IEEE 802.15.4, supports a maximum payload size of up to 2047 bytes with using almost the same PHY layer techniques as IEEE 802.15.4 [43].

**Figure 4:**
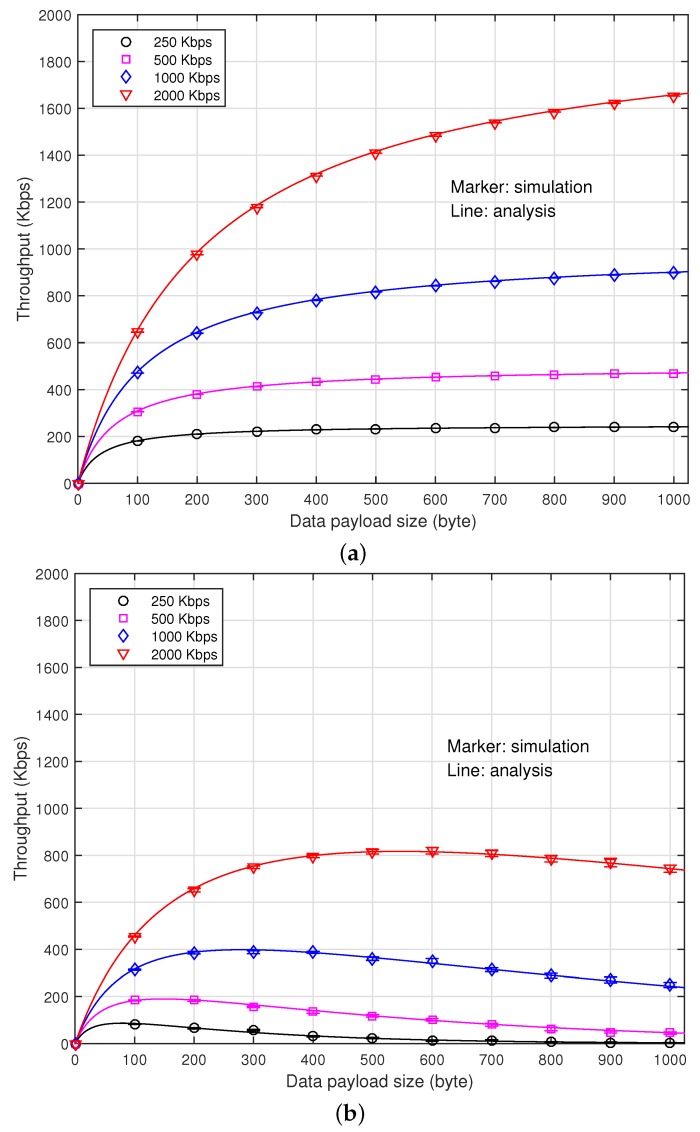
Throughput according to the payload size. (**a**) When *ρ* = 0; (**b**) When *ρ* = 0.2.

Figure 4 depicts the data throughput according to data payload size when the channel occupancy of the interference signal, *ρ*, is zero and 0.2. The error bar represents the standard deviation of the simulation result. To observe the impact of the channel occupancy of interference signal on the performance, we assume that $f_{D}=0\mathrm{Hz}$. We also assume that the SNR is high enough so that the transmitter can employ all of the transmission rates. It can be seen that when ρ=0, the data throughput increases indifferently from the transmission rate as the payload size increases, which is mainly due to the decrease of the header signaling overhead. When ρ=0.2, however, there exists a payload size maximizing the data throughput at each transmission rate. It is mainly due to the fact that the use of a larger payload size may become more susceptible to the collision. It can also be seen that the analytical results agree very well with the simulation results.

**Figure 5:**
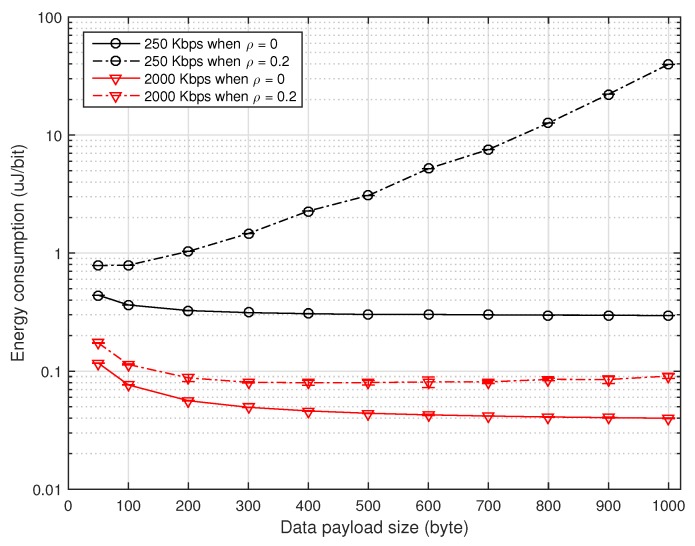
Energy consumption according to the payload size.

Figure 5 depicts the energy consumption (in uJoule/bit) of the WSN transmitter and receiver according to the data payload size when ρ=0 and 0.2, which is measured from the power consumption for the transmission and reception of all packets and the power consumption during idle listening (i.e., waiting a packet), as well. It can be seen that when ρ=0, the energy consumption somewhat decreases as the payload size increases mainly due to the increase of the throughput. Note that the energy consumption E=PavgPavgSS may increase as the payload size increases, where $P_{avg}$ denotes the average power consumption. When ρ=0.2, however, there exists a payload size that minimizes the energy consumption, which is slightly different from the one that maximizes the throughput. This is mainly due to the fact that $P_{avg}$ varies with the payload size. It can also be seen that the use of a higher transmission rate considerably reduces the power consumption, implying that the transmission rate should be adjusted according to the channel condition.

**Figure 6:**
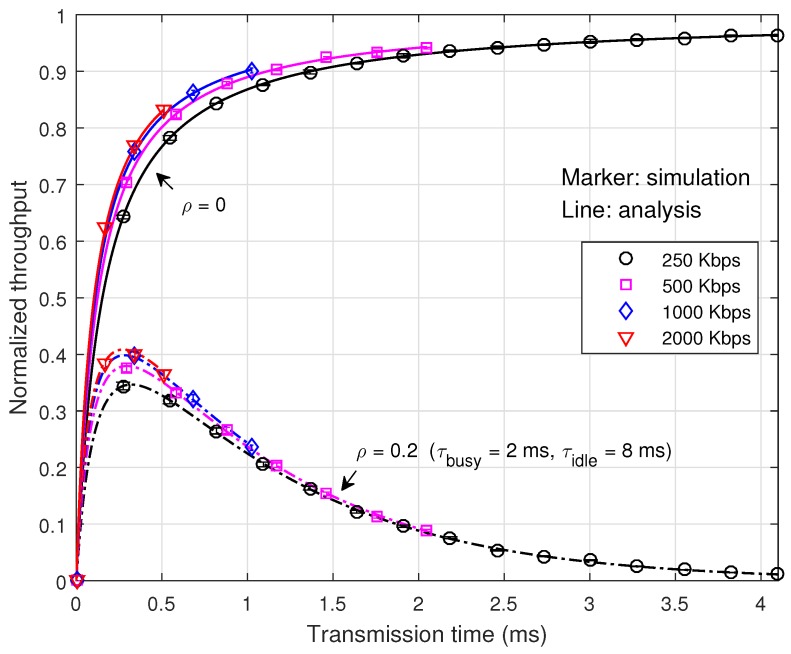
Normalized throughput according to the transmission time.

Figure 6 depicts the normalized throughput according to the transmission time. It can be seen that the normalized throughput and the optimum transmission time are quite affected by the channel occupancy of the interference signal, but little by the transmission rate. Note that the data throughput and the optimum payload size depend on the transmission rate. This property makes it desirable to adjust the transmission time according to the interference condition and the transmission rate according to the channel condition.

**Figure 7:**
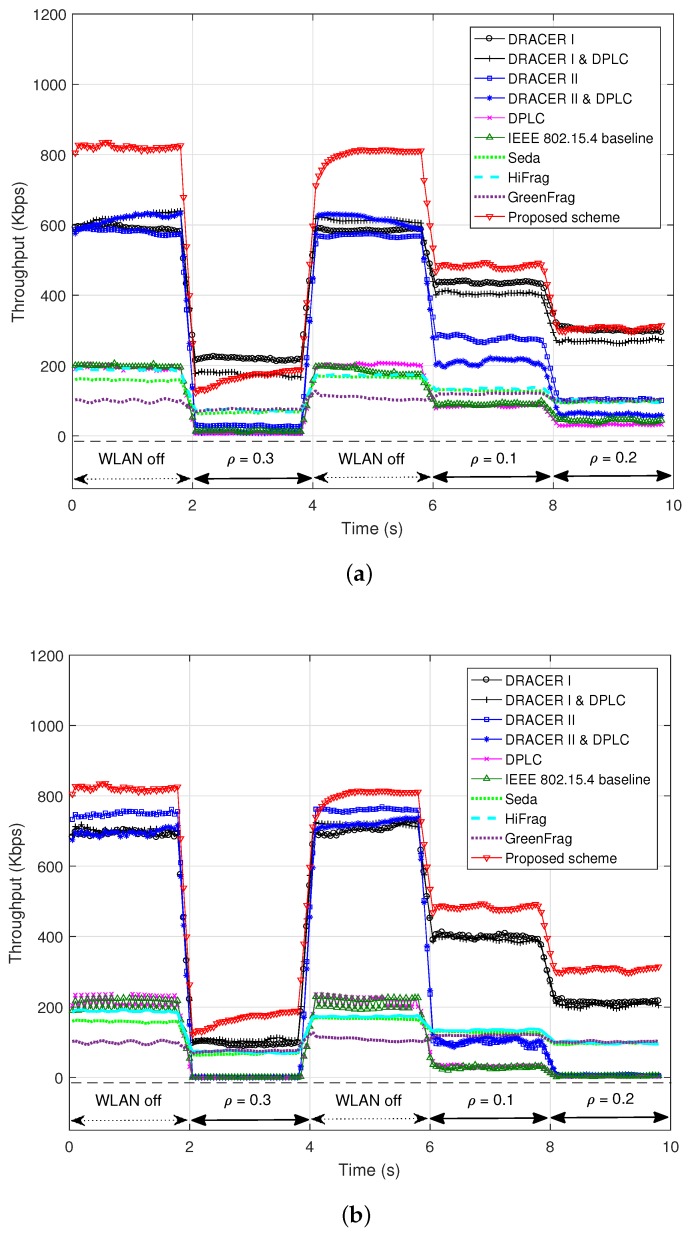
Throughput according to the change of interference. (**a**) When *L* = 300 bytes; (**b**) When *L* = 1000 bytes.

Figure 7 depicts the data throughput according to the channel occupancy of the interference signal when the SNR is 8 dB and the maximum Doppler frequency is 0.1 Hz, where the IEEE 802.15.4 baseline and DRACER use a fixed payload size of 300 or 1000 bytes, DPLC uses an initial payload size of 300 or 1000 bytes and adjusts it according to the performance and Seda, HiFrag and GreenFrag use their own frame structure proposed in their works, whereas the proposed scheme determines the initial payload size by estimating the average idle period of interference and then adjusts it according to the throughput performance. It can be seen that the IEEE 802.15.4 baseline provides very poor throughput performance even with the use of a large payload size (i.e., 300 or 1000 bytes) mainly due to the use of a low fixed transmission rate. It can also be seen that DRACER can improve the throughput performance by adjusting the transmission rate, but it may suffer from performance degradation with the use of a small fixed payload size in the absence of interference, which is mainly due to the transmission inefficiency, or with the use of a large fixed payload size in the presence of interference, which is mainly due to the increase of packet collision. We consider two DRACER schemes; DRACER I, which transmits packets at the highest rate regardless of transmission failure, and DRACER II, which adjusts the transmission rate in response to transmission failure. It was reported that these schemes are effective in an interference and a channel fading environment, respectively [22]. However, it can be seen that DRACER I and II make little difference on the transmission performance. This is mainly because they do not consider the effect of the payload size in the presence of interference, yielding inefficient use of white space. It can also be seen that DPLC can little improve the throughput performance even with the use of DRACER. This is mainly because it does not consider the effect of the transmission rate adjustment with a fixed step size of 10 bytes regardless of the transmission rate [23], yielding inefficient use of white space and slow adaptation of the payload size. It can also be seen that the proposed scheme significantly outperforms the other schemes by adjusting both the transmission rate and the transmission time in response to the change of interference and channel condition. It can also be seen that the PPR schemes (i.e., Seda, HiFrag and GreenFrag) may provide throughput improvement over the IEEE 802.15.4 baseline in the presence of co-channel interference. This is mainly because they partition the data packet into a number of small blocks and adapt the block size based on the transmission performance, which may provide robustness to co-channel interference. It can also be seen that they may outperform DPLC, which is mainly due to fast adaptation of the block size. However, PPR schemes may severely suffer from the presence of co-channel interference, mainly due to the frequent loss of recovery frames. It can also be seen that their performances are limited mainly due to the use of a low fixed transmission rate, fixed frame structure and small maximum payload size. It may not be easy for the PPR schemes to increase the maximum payload size and the transmission rate since the computational complexity may considerably increase as the number of blocks for the packet partitioning increases.

**Figure 8:**
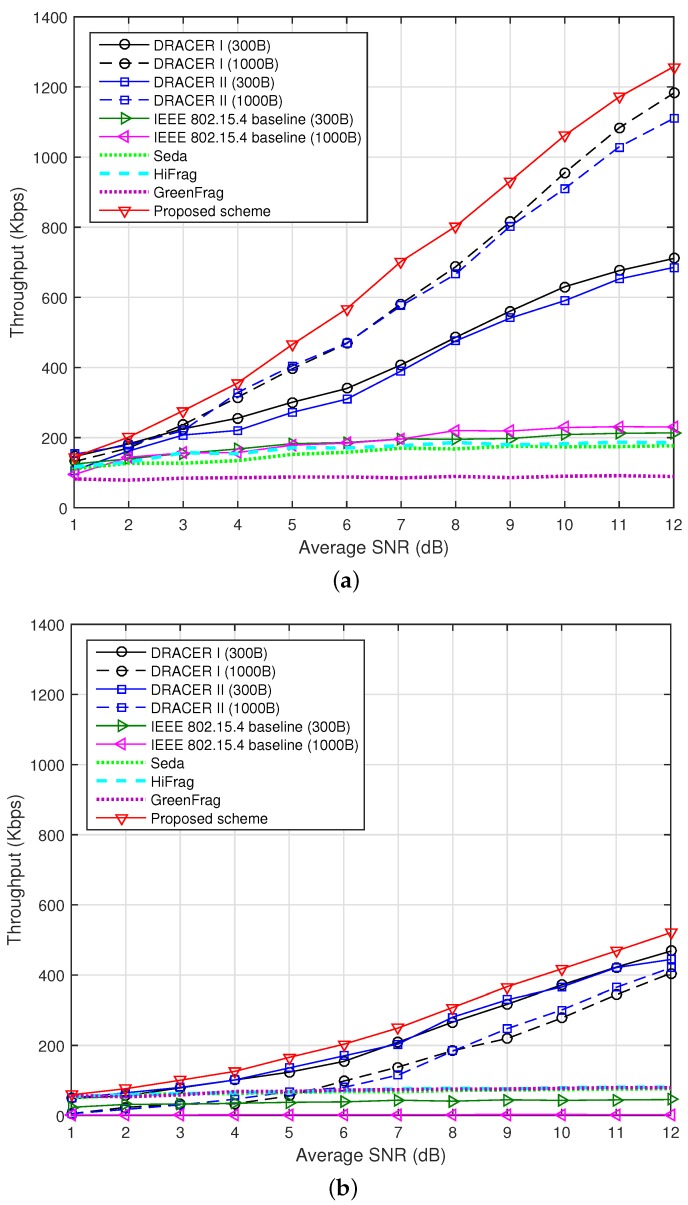
Throughput according to the SNR. (**a**) When *ρ* = 0; (**b**) When *ρ* = 0.2.

Figure 8 depicts the throughput according to the SNR when ρ=0 and 0.2. Since DPLC does not provide noticeable performance improvement over the IEEE 802.15.4 baseline and DRACER, we hereafter consider the performances of the IEEE 802.15.4 baseline and DRACER for the clarity of description. It can be seen that DRACER can improve the performance of the IEEE 802.15.4 baseline by using a large payload size in the absence of interference and a small payload size in the presence of interference. However, there is no proposed strategy when to employ DRACER I and II and how to adjust the payload size together in the presence of interference and channel fading. It can also be seen that the proposed scheme can significantly improve the throughput performance by independently adjusting the transmission rate and the transmission time according to the interference characteristics and channel condition, respectively. It can be seen that GreenFrag provides poorer throughput performance than HiFrag, although GreenFrag is a combination of HiFrag and transmit power adaptation, where it uses a transmit power level of 0, −3, −7, −15 or −25 dBm. When GreenFrag confirms good transmission performance, it reduces the transmit power without consideration of the channel condition. This may cause a ping-pong effect, seriously deteriorating the throughput performance. In fact, GreenFrag may not work well unless the SNR is sufficiently high (e.g., >30 dB).

**Figure 9:**
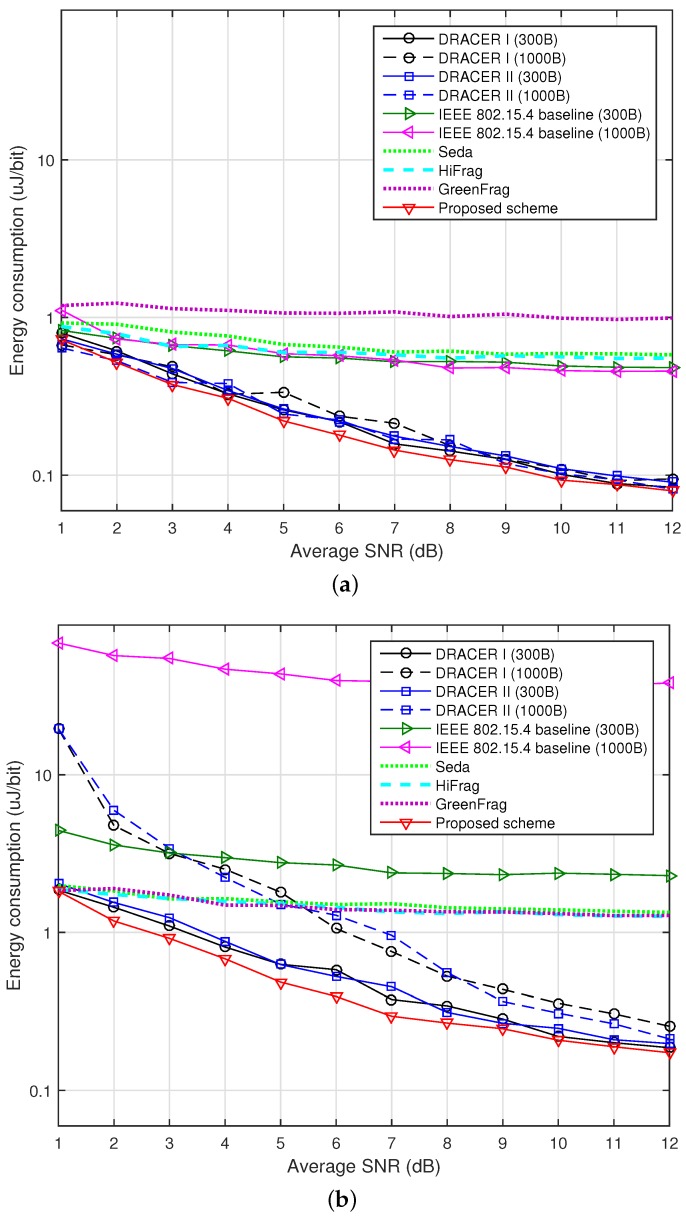
Energy consumption according to the SNR. (**a**) When *ρ* = 0; (**b**) When *ρ* = 0.2.

Figure 9 depicts the energy consumption (in uJoule/bit) according to the SNR when ρ=0 and 0.2. The power consumption of GreenFrag is measured at various power levels [28]. It can be seen that the power consumption increases when the SNR decreases or the channel occupancy of the interference signal increases, which is mainly due to the increase of transmission failure. It can also be seen that the proposed scheme reduces the power consumption by adjusting the transmission rate and the transmission time. However, the gain in power consumption is somewhat marginal. It is mainly because the use of a larger payload size in the absence of interference may increase the data throughput and the average power consumption, as well.

Table 2 summarizes the transmission delay according to the SNR when ρ=0 and 0.2, where $T_{\mathrm{period}}=983.04\mathrm{ms}$, $T_{\mathrm{comm}}=0.5\;T_{\mathrm{period}}$ and $L_{\mathrm{bulk}}=65\mathrm{Kbytes}$. It can be seen that the transmission delay decreases when the SNR increases or the channel occupancy of the interference signal decreases, which is mainly due to the increase of the data throughput. It can also be seen that the proposed scheme and DRACER significantly outperform the other schemes, which is mainly due to the transmission rate adjustment. It can also be seen that the proposed scheme can reduce the transmission delay further than DRACER both in the absence and the presence of interference. This is mainly because the proposed scheme can adjust both the transmission rate and the payload size to maximize the data throughput in response to the change of operation environments.

**Figure 10:**
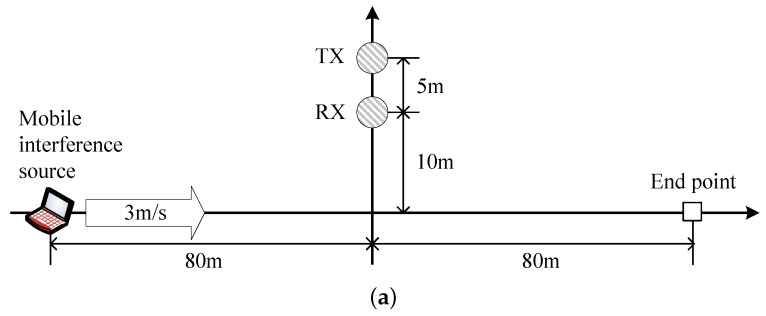

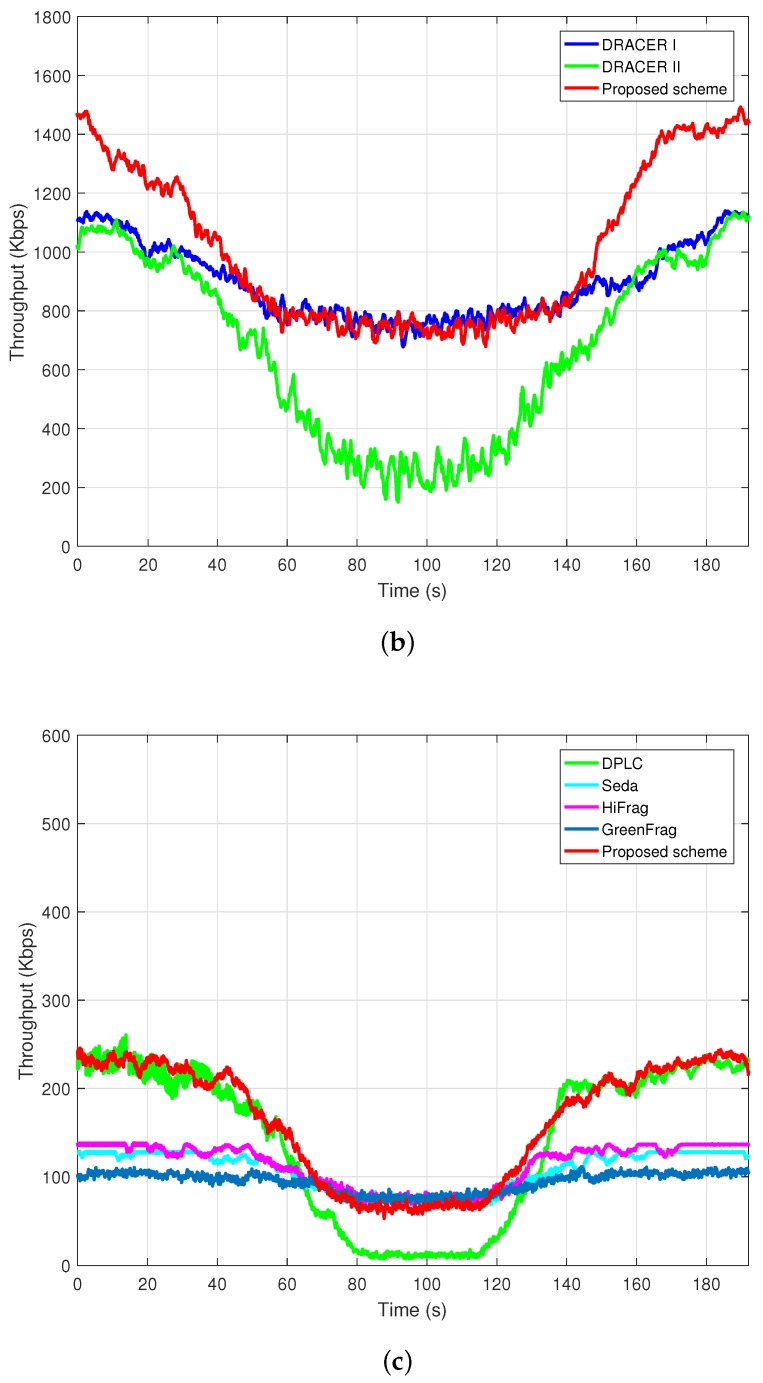
Transmission performance in the presence of mobile interference source. (**a**) Simulation scenario (without any shadowing); (**b**) Throughput according to time when the maximum *R* is 2 Mbps; (**c**) Throughput according to time when the maximum *R* is limited to 250 Kbps.

Figure 10b,c depicts the throughput in the presence of the interference signal with ρ=0.2 and a mobility of 3 km/h, as illustrated in Figure 10a, where the SNR slowly changes with a value of 35∼40 dB, but the SINR changes from −20 to 20 dB when the maximum transmission rate is limited to 2 Mbps and 250 Kbps, respectively. Figure 10b depicts the performance of the proposed scheme and DRACER since they outperform the other schemes. It can be seen that DRACER I outperforms DRACER II, which is mainly because it keeps the highest rate indifferently from the transmission failure. When the SNR is not high enough to employ the highest rate, however, DRACER I may not outperform DRACER II (as shown in Figure 7). It can also be seen that the proposed scheme outperforms DRACER I and II by adjusting both the transmission rate and the payload size, maximally exploiting the white space of the interference signal. The performance gap between the proposed scheme and DRACER I increases as the SNR decreases. Figure 10c depicts the performance of the proposed scheme without adjustment of the transmission rate, DPLC and the PPR schemes. It can be seen from Figure 10c that DPLC provides poor throughput performance, which is mainly due to the slow adaptation of the payload size. It can also be seen that the performance of PPR schemes is limited mainly due to the use of a fixed frame structure and small maximum payload size. It can also be seen that the proposed scheme outperforms the other schemes, which is mainly due to the fast adaptation of the payload size with a large maximum payload size. Note that it may not be easy for the PPR schemes to increase the maximum payload size since the computational complexity may considerably increase as the number of blocks for the packet partitioning increases.

## 6. Conclusions and Future Works

In this paper, we have considered the performance improvement of low-power WSNs in practical operation environments, where strong co-channel interference may exist. We have investigated the throughput performance of WSNs in the presence of interference and then designed a transmission scheme to maximize it. We have considered the adjustment of transmission rate and transmission time in response to the change of operation environments. The analytic and simulation results show that the proposed scheme significantly improves throughput, while preserving energy efficiency even in the presence of severe interference. For channel diversity, it may be helpful to use a dynamic channel hand-off mechanism (e.g., [40]). The proposed scheme can directly be applied to IEEE 802.15.4e DSME MAC [44] and 15.4g PHY-based [43] WSNs. It can also be applied to a tree or mesh topology with an appropriate link scheduling scheme that can provide robustness to collision between multiple communication links [45,46].

## Figures and Tables

**Table 1:** Simulation parameters. SHR, synchronization header; PHR, physical layer packet header; MHR, MAC layer packet header; CCA, clear channel assessment.

Parameters	Values
SHR and PHR sizes $\left(L_{\mathrm{shr}},L_{\mathrm{phr}}\right)$	5 bytes, 1 byte
MHR size of data and ACK $\left(L_{\mathrm{mhr}}^{data},L_{\mathrm{mhr}}^{ack}\right)$	9 bytes, 5 bytes
Transmission rate of SHR and PHR $\left(R_{\mathrm{base}}\right)$	250 Kbps
Transmission rate of MHR and payload (R)	250/500/1000/2000 Kbps
(variable rate transceiver structure)	[22,41,42]
Path loss model	Channel model in [35]
Transmit power	0 dBm
Turnaround time (δ)	192 us
Channel sensing interval $\left(T_{s}\right)$	320 us
Total data size $\left(L_{\mathrm{bulk}}\right)$	65 Kbytes
Power consumption during transmission	49.9 mW
Power consumption during reception	56.5 mW
Power consumption in the idle mode	1.2 mW
Transmission failure threshold $\left(N_{\mathrm{fail}}\right)$	3
Payload adjustment threshold (η)	1.2
Step size $\left(\Delta_{1},\Delta_{2}\right)$	2 (no unit), 320 us
Window size (W)	10
Payload size limit $\left(L_{\min},L_{\min}\right)$	20 bytes, 1024 bytes
Period of the frame $\left(T_{\mathrm{period}}\right)$	983.04 ms
Length of the data communication interval $\left(T_{\mathrm{comm}}\right)$	491.52 ms
Simulation time slot	40 us
Maximum Doppler frequency $\left(f_{D}\right)$	0.1 Hz
WLAN packet length $\left(\tau_{\mathrm{busy}}\right)$	2 ms
WLAN idle state distribution	Exponential
WLAN CCA type	Carrier sensing
WLAN transmit power	17 dBm

**Table 2:** Transmission delay according to the SNR when ρ=0 and 0.2 (unit: s). DRACER, dynamic rate adaptation and control for energy reduction; HiFrag, hybrid frame fragmentation scheme.

SNR	2 dB	2 dB	6 dB	6 dB	12 dB	12 dB
ρ	0	0.2	0	0.2	0	0.2
DRACER I (300 B)	5.9	19.0	3.9	7.0	2.0	3.0
DRACER I (1000 B)	6.9	46.0	2.9	11.0	1.0	3.0
DRACER II (300 B)	6.9	17.0	3.9	7.0	2.0	3.0
DRACER II (1000 B)	5.9	61.1	2.9	14.0	1.0	3.0
15.4 baseline (300 B)	7.9	34.1	5.9	28.1	4.9	24.0
15.4 baseline (1000 B)	7.9	690.4	5.9	396.8	4.9	396.8
Seda	8.8	19.0	6.9	16.0	5.9	14.0
HiFrag	8.8	18.0	6.9	15.0	5.9	14.0
GreenFrag	13.8	20.0	12.8	15.0	11.8	14.0
Proposed scheme	5.9	14.0	2.0	6.0	1.0	3.0

## Appendix A. Minimum SINR for IEEE 802.15.4 Communications

The minimum SINR to achieve a desired packet error rate of $p_{s}$ with the use of packet size *L* can be represented as:

(A1) $$\hat{\gamma }=b^{-1}\left(1-p_{s}^{1/L}\right)$$

where *b* denotes the BER and $b^{-1}$ denotes its inverse. The BER of IEEE 802.15.4 PHY can be represented as [35]:

(A2) $$b\left(\gamma \right)=\frac{8}{15}\times \frac{1}{16}\times \sum_{k=2}^{16}{\left(-1\right)}^{k}\left(\begin{array}{c} 16\\ k \end{array}\right)e^{20\gamma \left(\frac{1}{k}-1\right)}.$$

Since the BER is a decreasing function of SINR, it can be seen that $b\left(\gamma \right)$ has an inverse function. However, it may not be feasible to obtain the inverse function of (A2) in a closed form. A numerical method can be applied to approximately calculate (A1). Table A1 summarizes the minimum SINR according to the packet size *L*.

**Table A1:** Minimum SINRs for IEEE 802.15.4 communications.

*L*	20 Bytes	40 Bytes	60 Bytes	80 Bytes	100 Bytes	120 Bytes
$\hat{\gamma }$	0.40 dB	0.68 dB	0.83 dB	0.93 dB	1.01 dB	1.07 dB

## Appendix B. Derivation of Equation (2)

It can be shown that the packet error rate (PER) without channel coding in the presence of interference can be represented as:

(B1) $$p=1-{\left(1-b\left(\gamma_{\mathrm{SNR}}\right)\right)}^{L_{\mathrm{pkt}}-L_{\mathrm{int}}}{\left(1-b\left(\gamma_{\mathrm{SINR}}\right)\right)}^{L_{\mathrm{int}}}$$

where $b\left(\gamma \right)$ denotes the BER at an SNR of *γ*, $\gamma_{\mathrm{SNR}}$ denotes the SNR in the absence of interference, $\gamma_{\mathrm{SINR}}$ denotes the SINR in the presence of interference signal, $L_{\mathrm{pkt}}$ denotes the bit size of a packet and $L_{\mathrm{int}}\left(\leq L_{\mathrm{pkt}}\right)$ denotes the number of bits colliding with the interference signal. Since $L_{\mathrm{int}}$ can randomly be changed, the average PER can be represented as:

(B2) $$\begin{array}{ccc} \bar{p} & = & E_{L_{\mathrm{int}}}\left[p\right]\\ & = & 1-\left(1-p_{s}\right)E_{L_{\mathrm{int}}}\left[K^{L_{\mathrm{int}}}\right] \end{array}$$

where $p_{s}=1-{\left(1-b\left(\gamma_{\mathrm{SNR}}\right)\right)}^{L_{\mathrm{pkt}}}$ and K=1-bγSINR1-bγSINR1-bγSNR1-bγSNR. Note that 0.5<K<1 since $0<b\left(\gamma_{\mathrm{SNR}}\right)<0.5$, $0<b\left(\gamma_{\mathrm{SINR}}\right)<0.5$ and $b\left(\gamma_{\mathrm{SNR}}\right)<b\left(\gamma_{\mathrm{SINR}}\right)$. Without information on the probability distribution function of $L_{\mathrm{int}}$, it may not be easy to calculate the expectation. For ease of calculation, define a random variable *φ* by:

(B3) $$\varphi =\left\{\begin{array}{c} 1;L_{\mathrm{int}}=0\\ 0;L_{\mathrm{int}}>0 \end{array}\right.$$

It can be shown from $\varphi \leq K^{L_{\mathrm{int}}}$ for all $L_{\mathrm{int}}$ that:

(B4) $$\begin{array}{ccc} \tilde{p} & = & 1-\left(1-p_{s}\right)\left(1-p_{c}\right)\\ & \geq & 1-\left(1-p_{s}\right)E_{L_{\mathrm{int}}}\left[K^{L_{\mathrm{int}}}\right]=\bar{p} \end{array}$$

where $p_{c}$ denotes the probability of transmission failure assuming that the packet transmission is failed whenever the collision occurs and equals $1-E_{L_{\mathrm{int}}}\left[\varphi \right]$. The assumption may be valid if SINR is sufficiently low. Table B1 summarizes pairs of $\gamma_{\mathrm{SINR}}$ and $L_{\mathrm{int}}$ yielding $K^{L_{\mathrm{int}}}<0.01$ for IEEE 802.15.4 communications.

**Table B1:** Conditions making $K^{L_{\mathrm{int}}}$ < 0.01 for IEEE 802.15.4 communications.

$b\left(\gamma_{\mathrm{SINR}}\right)$	0.01	0.1	0.2	0.3	0.4	0.44
$\gamma_{\mathrm{SINR}}$	−2.5 dB	−5.6 dB	−7.5 dB	−9.5 dB	−12.5 dB	−14.6 dB
$L_{\mathrm{int}}$	57.3 bytes	5.5 bytes	2.6 bytes	1.6 bytes	1.1 bytes	<1 byte
